# Safety and immunogenicity of the yellow fever vaccine for patients with end-stage renal disease

**DOI:** 10.1590/2175-8239-JBN-2023-0202en

**Published:** 2024-08-26

**Authors:** Jesiree Iglésias Quadros Distenhreft, Dinair Couto-Lima, Cecilia Siliansky de Andreazzi, Juliana Feu Rosa Carrera Thomazini, Lauro Monteiro Vasconcellos, Aloísio Falqueto, Weverton Machado Luchi

**Affiliations:** 1 Universidade Federal do Espírito Santo, Faculdade de Medicina, Hospital Universitário Cassiano Antônio Moraes, Serviço de Nefrologia, Vitória, ES, Brazil.; 2 Instituto Oswaldo Cruz, Laboratório de Mosquitos Transmissores de Hematozoários, Rio de Janeiro, RJ, Brazil.; 3 Instituto Oswaldo Cruz, Laboratório de Biologia e Parasitología de Mamíferos Silvestres Reservatórios, Rio de Janeiro, RJ, Brazil; 4 Universidad Complutense de Madrid, Faculdad de Ciencias Biológicas, Departamento de Biodiversidad, Ecología y Evolución, Madrid, España.; 5 Clínica Nefrológica de Colatina, Colatina, ES, Brazil.; 6 Universidade Federal do Espírito Santo, Centro de Ciências da Saúde, Programa de Pós-Graduação em Doenças Infecciosas, Vitória, ES, Brazil.

**Keywords:** Renal Insufficiency, Chronic, Efficacy, Antibodies, Neutralizing, Safety, Yellow Fever Vaccine

## Abstract

**Introduction:**

In December 2016, an outbreak of sylvatic yellow fever (YF) occurred in the non-endemic areas of the south-eastern region of Brazil. The immune response to the yellow fever vaccine and its safety in individuals with chronic kidney disease (CKD) living in YF-endemic regions are not thoroughly understood. The objective of this study is to assess the incidence of adverse events and the serological response after primary vaccination with the 17DD-YF vaccine in CKD patients undergoing dialysis.

**Methods:**

This was a multicenter, retrospective cohort study involving 223 individuals with CKD who were on dialysis after primary vaccination against YF. Clinical and epidemiologic characteristics were collected and the vaccine adverse event (VAE) were assessed. Around 35 months after vaccination, the serological response was evaluated in 71 (32%) patients using neutralization tests.

**Results:**

No serious VAE occurred in any patient. Local reactions were reported in 13 individuals (5.8%), while 6 (2.7%) reported generalized systemic reactions and 205 (91.9%) did not display any VAE. No clinical or epidemiologic characteristic predicted the occurrence of VAE. Adequate serological response was found in 38% of participants and none of the clinical or epidemiological characteristics were associated with immunogenicity.

**Conclusion:**

The outcomes of our study suggest that the yellow YF vaccine is well-tolerated in CKD patients undergoing dialysis, but it does not induce adequate immune response. Future research should focus on evaluating both cellular and humoral immune responses following administration of various doses of the YF vaccine.

## Introduction

In December 2016, an outbreak of sylvatic yellow fever (YF) started in non-endemic and densely populated areas in south-eastern Brazil, mainly on the coast. YF is a disease with high mortality and there is no specific treatment. Vaccination is the most effective preventive measure against the infection^1^. A vaccination campaign has therefore been launched in the affected areas.

The vaccine against YF that is currently used in Brazil is the 17DD live-attenuated vaccine from Oswaldo Cruz Foundation Institute of Immunobiological Technology (Bio-Manguinhos). About 10 days after vaccination, at least 80% of people achieve adequate immune titers, and after 30 days, more than 99% of people also achieve this. The current recommendation is that one dose is sufficient to provide lifelong protection^2^. However, this response may be reduced in immunocompromised individuals^3^.

Adverse reactions to the YF vaccine, typically mild, may encompass symptoms such as headache, myalgia, low-grade fever, and discomfort at the injection site, affecting approximately 4% of vaccinated individuals. Although generally safe, the YF live attenuated vaccine can, in rare cases, lead to severe conditions such as hypersensitivity, acute neurologic disease, or vaccine-associated viscerotropic disease. Contraindications to the vaccine include children under six months of age, pregnant women, immunocompromised individuals, history of thymus disease, and individuals who have experienced anaphylactic reactions to vaccine components^4,5^.

Considering that it is a live attenuated virus vaccine, the YF vaccine is not recommended for immunocompromised individuals due to the risk of major adverse events. However, given the possibility of urban occurrence in Brazil, as occurs in Africa, it is necessary to evaluate the safety of the YF vaccine for immunocompromised people in endemic areas. The high density of *Aedes aegypti* infestation, the increase in the number of sylvatic YF cases, and the low vaccination coverage in Brazil favor the re-emergence of the disease in urban settings^6^.

The YF vaccine should be administered to patients with CKD undergoing dialysis if there are no contraindications^7^. Immunity in CKD is influenced by factors such as uremic toxins, malnutrition, chronic inflammation, alterations in the vitamin D-parathyroid hormone axis, and therapeutic dialysis. The coexistence of chronic immune activation and suppression in CKD may render individuals more susceptible to adverse effects of the YF vaccine and impaired responses to vaccination^8,9,10^.

During the YF outbreak in Brazil, many individuals with CKD were vaccinated due to the risk of YF. The objective of this study was to evaluate the occurrence of adverse events and serological response after the 17DD-YF primary vaccination in patients with CKD on dialysis.

## Methods

### Study Design

This multicenter retrospective cohort study involved individuals with CKD who received the YF vaccine while undergoing renal replacement therapy. Between January 2020 and August 2021, we selected patients with CKD on dialysis in institutions in the metropolitan region of Vitoria and Colatina, Espírito Santo, Brazil, which was affected by the YF outbreak. All participants had previously received the 17DD-YF primary vaccination (Bio-Manguinhos-FIOCRUZ). The majority of participants were vaccinated during the 2017 Brazilian YF vaccination campaign. Some individuals had already received the vaccine before 2017 because they lived in or traveled to endemic regions. This study was approved by the Research Ethics Committee of the Centro de Ciências da Saúde of the Universidade Federal do Espírito Santo (CAAE 24851419.9.0000.5060). All study participants signed a consent form after all doubts had been resolved by the researchers.

The study population consisted of adults of both sexes over 18 years of age, who were vaccinated against YF while undergoing renal replacement therapy (peritoneal dialysis or hemodialysis). Only vaccination verified by a vaccination card was considered.

The medical institutions that participated and the number of patients served by these services of renal replacement therapy were: Instituto de Doenças Renais (Metropolitan Region of Vitória, 341 patients), Hospital Universitário Antônio Cassiano de Moraes (Vitória, 99 patients), Casa de Saúde de Santa Maria (Colatina, 133 patients), and Clínica Nefrológica de Colatina (Colatina, 250 patients). Individuals who were vaccinated before becoming dialysis patients were not included in this study.

All participants were interviewed and their vaccination charts and medical and laboratory examination records for the month following vaccination were reviewed. From this population, serum samples from 71 individuals (32%) underwent plaque reduction neutralization testing (PRNT).

### Safety Evaluation

Structured interviews were conducted during the hemodialysis sessions or on the same day as the routine examinations to evaluate the epidemiological and clinical data as well as the side effects of the vaccine. Additionally, patients’ medical records in the month following vaccination were also reviewed.

Patients on renal replacement therapy undergo monthly blood collection for liver enzyme measurement and annual for hepatitis B, hepatitis C, and HIV serology. The notation of clinical manifestations and laboratory test results are available in dialysis service records.

Adverse events were classified by extent (local versus systemic) and type (general manifestations, hypersensitivity, neurological, and viscerotropic disorders) according to the Manual of Epidemiological Surveillance of Adverse Events Following Immunization of the Brazilian Ministry of Health. All symptoms in the region where the vaccine was applied were considered local adverse events, such as pain, edema, erythema, hyperemia, lumps, or abscesses. General systemic manifestations included fever, myalgia, cephalea, arthralgia, weakness, abdominal pain, nausea, and tremor. Hypersensitivity, neurological, and viscerotropic disorders were considered major adverse events.

### Testing for Immunogenicity

In February 2020, in order to detect neutralizing antibodies, 5 mL of peripheral blood was collected from 71 participants and stored in vacuum tubes without coagulants. The blood was centrifuged and the serum was stored at –80 °C. The samples were processed at the Laboratório de Mosquitos Transmissores de Hematozoários (LATHEMA, Fundação Oswaldo Cruz – Fiocruz – RJ).

The rates of anti-YF neutralizing antibodies were assessed through PRNT, a reference method to evaluate post-vaccination immunogenicity. Neutralization titers (NT) of 1:10 and 1:20 were used as cut-off values in accordance with studies on the passive immunization of hamsters and evidence of protective titers in other arboviruses, such as the Japanese encephalitis virus^11,12^. For this study, titers above 1:10 were used for screening and titers above than 1:20 were used as correlates of immunity.

Normally, the PRNT is only performed with the vaccine strain. However, we have created a model to compare the attenuated vaccine virus with the wild virus. In our study, we tested the samples with two wild strains in addition to the vaccine virus: IEC-4408 (YFV-4408) and ES-504/BRA/2017. Both viruses were isolated from the serum of non-human primates during epidemics in Brazil, first in 2008 in Rio Grande do Sul and then in 2017 in Espírito Santo.

Initially, the 71 samples underwent screening using the 17DD vaccine strain. Samples exhibiting neutralization above 90% at a dilution of ≥1:10 (indicative of detectable neutralizing antibodies) were subjected to progressive dilution, ranging from 1:20 to equal to or greater than 1:640. An immunological titer was considered adequate when NT was greater than or equal to 1:20. Only samples with an adequate titer were subsequently challenged with the wild-type strains.

### Data Analysis

After data collection, the frequency of adverse events and their nature were verified and the characteristics that could predict the occurrence of adverse events were evaluated. The univariate analysis was conducted with the chi-square test for categorical variables in the Past software and with simple logistic regression for quantitative variables in the BioEstat software. After, multivariate analysis was performed in the R software by multiple logistic regressions. The multiple correspondence analysis was done to exhibit the association between predictive factors and the occurrence of adverse events.

To determine whether there was any predictive characteristic for the serological response, a multiple correspondence analysis was first performed. Then, the most significant characteristics were submitted to multivariate analysis in R software by multiple logistic regression. And then the likelihood ratio test was performed.

The various associations were expressed by chi-square rates, with Z as independent variants and odds ratio determined by the logistic regression model, and respective 95% confidence interval. In all cases, values of p <0.05 were considered statistically significant.

## Results

### Adverse Events

A total of 223 adults undergoing renal replacement therapy participated in the study. The age of the subjects ranged from 19 to 87 years (mean, 51.13 and median, 52 years); 61.9% of them were men. The most common underlying conditions was high blood pressure (69.1%), diabetes mellitus (25.6%), and heart failure (5.4%). Among individuals with a history of kidney transplant, two were still using immunosuppressive drugs (prednisone, cyclosporine, and mycophenolate). Additionally, four participants were using immunosuppressive drugs due to rheumatic diseases (prednisone, cyclosporine, and mycophenolate). The other characteristics of the studied population are described in Table 1.

**Table 1:** Characteristics of the study population.

Characteristics	YF vaccine safety Total (223)	PRNT evaluationTotal (71)
Male gender, n (%)	138 (61.9)	37 (52.1)
Age, years, mean ± SD	51. (14.1)	51.08 (13.2)
Race, n (%)		
White	89 (39.9)	23 (32.39)
Black	67 (30.1)	20 (28.2)
Brown/Mixed-race	66 (29.6)	28 (39.4)
Yellow	1 (0.4)	0
Indigenous	0	0
Tobacco use, n (%)	44 (19.7)	9 (12.7)
Alcoholism, n (%)	25 (11.2)	10 (14.1)
Comorbidities, n (%)		
High blood pressure	154 (69.1)	54 (76.1)
Diabetes mellitus	57 (25.6)	20 (28.2)
Cancer^a^	4 (1.8)	0 (0)
Rheumatic diseases	5 (2.2)	2 (2.8)
Heart failure	12 (5.4)	6 (8.4)
HIV	0	0
Hepatitis C	4 (1.8)	2 (2.8)
Hepatitis B	4 (1.8)	0 (2.8)
Chronic kidney disease etiology, n (%)		
Glomerulopathies	44 (19.7)	16 (22.5)
Diabetes mellitus	47 (21.1)	18 (25.3)
High blood pressure	55 (24.7)	17 (23.9)
Cystic disease	8 (3.6)	5 (7.1)
Congenital disease	13 (5.8)	2 (2.8)
Others	18 (8.1)	10 (14.1)
Unknown	38 (17.1)	5 (7.1)
Hemodialysis, n (%)	211 (94.6)	66 (93)
Time on dialysis, years, median (IQR)	9.5 (6.5)	10 (4.8)
Peritoneal dialysis, n (%)	12 (5.4)	5 (7.1)
Time on dialysis, years, median (IQR)	7.2 (5)	11.9 (7)
Previous kidney transplant, n (%)	16 (7.2)	6 (8.5)
Use of immunosuppressive drugs^b^, n (%)	6 (2.7)	1 (1.4)

Abbreviations: SD, standard deviation; IQR, interquartile range.

Notes:

a Cancers found: skin (one), prostate (one), kidney (one), rectum (one).

b Prednisone, cyclosporine and mycophenolate.

Among this population, 205 individuals (91.9%) did not experience any vaccine adverse events (VAE). Adverse events, classified as either local or systemic manifestations, were observed in 18 individuals (8.1%). However, no one experienced a serious adverse event (Table 2).

**Table 2:** Adverse events (AE) following the yellow fever (YF) vaccination.

AE following vaccination	YF vaccine safety (223)n (%)
At least one AE	18 (8.1)
Local AE	13 (5.8)
Pain	13 (5.8)
Edema	0
Erythema	1 (0.4)
Systemic AE	6 (2.7)
Myalgia	2 (0.9)
Fever	3 (1.3)
Headache	2 (0.9)
Malaise	3 (1.3)
Abdominal pain	1 (0.4)
Severe AE	0
Hospitalization	1 (0.4)
Death	0
ALT Levels	218 (56.9)
0–30 U/L	4 (1.8)
31–50 U/L	1 (0.4
51–70 U/L	

Abbreviation: ALT = alanine aminotransferase test. Note: One of 223 individuals had both local and systemic AE. The hospitalized patient presented fever, malaise, abdominal pain, myalgia, and an increase in ALT levels.

One patient with no prior serological evidence of hepatitis B and C exhibited clinical manifestations of acute hepatitis, including fever, myalgia, and abdominal pain, along with elevated ALT levels, twice as high as the reference value, in the month following vaccination. Additionally, this individual was the only one requiring hospitalization, although they fully recovered from elevated transaminase levels within approximately two months, with no lasting medical consequences.

We evaluated the predictions for the occurrence of adverse events. In the univariate analysis, mixed race (χ^2^ = 16; p < 0.002) and glomerulopathy (χ^2^ = 7; p < 0.04) were associated with adverse events. After multivariate analysis, only “mixed race” remained as a risk factor for the occurrence of adverse events (Table 3).

**Table 3:** Factors predicting adverse events following vaccination in patients with chronic kidney disease undergoing dialysis.

Characteristics	Prediction of the occurrence of adverse factors							
	Univariate Analysis				Multivariate Analysis			
	N	OR	(95%CI)	p	N	OR	(95%CI)	p
Mixed-race	11	4.3	1.6–11.6	0.002	223	4	1.4–10.9	0.008
Use of immunosuppressive drugs	2	5	0.9–27.8	0.04	223	3.4	0.5–22.7	0.21
Glomerulopathy	44	2.9	1–7.9	0.033	223	2.5	0.8–7.3	0.099

Notes: Adjusted odds ratio for the risk of adverse events post-vaccination. The multiple logistic regression analysis estimated that mixed race would be associated with the occurrence of adverse events after vaccination.

**Figure 1:**
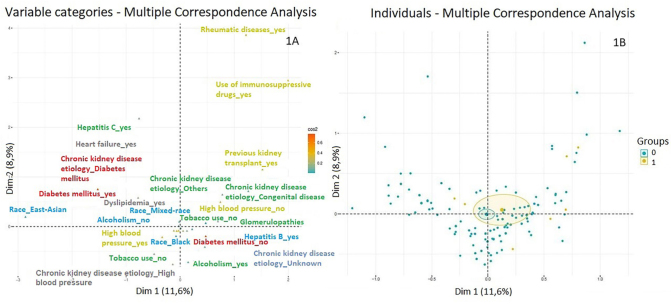
Assessment of the safety of the Yellow Fever vaccikne in Chronic Kidney Disease patients on dialysis (n = 223). **A** – Association between the variants included in the reduced multiple correspondence analysis model. The association between the characteristics of individuals is shown in the two dimensions above. We observed that, although there is some level of clustering between some characteristics, no defined groups are formed in opposition. **B** – Distribution of individuals according to the reduced multiple correspondence analysis. Individuals who had adverse events are represented in yellow, while individuals who had no adverse events are in blue. As shown, the blue circle is within the yellow circle, which suggests that there are no distinct groups.

The association between predictive factors and occurrence of VAE was examined using multiple correspondence analysis, as shown in Figure 1 (A and B). Two dimensions that collectively explain 20.5% of the variance were identified: the first dimension accounted for 8.9% of the variability, while the second explained 11.6%. Figure 1A shows the correlation between variables, while Figure 1B represents each individual participant in the study.

In Figure 1B, individuals who experienced adverse events are depicted in yellow, while those who did not are shown in blue. The characteristics of individuals are correlated with those illustrated in Figure 1A. Notably, individuals with adverse events are dispersed across the graph area, indicating a lack of direct connection between their characteristics and adverse events. The blue and yellow circles provide an approximation of each group’s distribution. Interestingly, the blue circle is contained within the yellow one, suggesting the absence of distinct groups.

### Immunogenicity

The PRNT was performed in patients who presented for blood sampling on the date proposed by the researchers, twelve patients from the Vitória metropolitan area and 59 patients from Colatina. The PRNT was performed in 71 participants approximately 35 months after vaccination, with a range of 20 to 82 months. Of these, 67 individuals (94.4%) were vaccinated in 2017, two (2.8%) in 2018, one (1.4%) in 2013 and one (1.4%) in 2016.

Detectable neutralizing antibodies (NT ≥ 1:10) were found in 27 participants (38%). These individuals also exhibited adequate neutralization titers (NT ≥ 1:20) for all three strains (vaccine virus, Es504, and 4408). The only patient that was using immunosuppressive drugs did not have NT ≥ 1:20.

**Figure 2:**
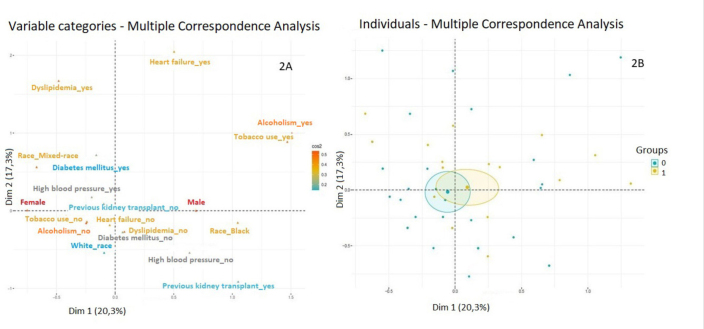
Assessment of the immunogenicity of the Yellow Fever vaccine in Chronic Kidney Disease patients on dialysis (n = 71). **A** – Association between the variants included in the reduced multiple correspondence analysis model. The association between the characteristics of individuals is evidenced in the two dimensions above. Although some clustering between some characteristics is visible, no defined groups are formed in opposition. **B** – Distribution of individuals according to the reduced multiple correspondence analysis. Individuals who had NT ≥1:20 are represented in yellow (Group 1), while individuals without serological response (Group 0) are shown in blue. The overlap of the two circles indicate the absence of statistical significance.

Following multiple correspondence analysis to identify the most representative categories (Figure 2A), a multivariate analysis was conducted using logistic regression, as shoswn in Table 4. High blood pressure was the only characteristic with a significant effect on seroconversion (coefficient = 2.1; p = 0.02), albeit with low explanatory power. However, despite the significance of this variable, the multivariate model did not provide much additional explanation, as it did not differ significantly from the null model (likelihood ratio test; p = 0.24). The same data are shown in Figure 2B, where the individuals who had a sufficient neutralization titer are shown in yellow, while those who did not are shown in blue. The characteristics of these individuals are correlated with those in Figure 2A. The blue and yellow circles represent an approximation of the distribution of each group. We observe an overlap between the two circles, indicating that there is no statistically significant variable.

**Table 4:** Predictors of serological response in patients with chronic kidney disease undergoing dialysis.

Variants	Coefficient	Standard error	Z rate	p-rate
Male sex	0.9	1.7	1.3	0.20
Black race	0.4	0.7	0.5	0.64
Brown or mixed race	0.1	0.8	0.2	0.85
Previous kidney transplant	0.1	0.8	0.1	0.96
Alcoholism	0.1	1.1	0.1	0.88
Tobacco use	0.9	1.0	1.0	0.32
HBP	2.1	0.9	2.4	0.02
DM	–0.5	0.9	–0.6	0.55
Heart failure	0.4	0.8	0.3	0.77
Dyslipidemia	0.2	1.2	0.2	0.84
Age	0.0	0.9	–1.3	0.18
Time on dialysis	0.0	0.0	0.7	0.47

Abbreviations: HBP: High Blood Pressure (CKD etiology); DM; Diabetes mellitus (CKD etiology). Note: High blood pressure appears to be a predictive factor of seroconversion (Coefficient = 2.1; p = 0.02).

## Discussion

In this study, we provided the first demonstration of the safety and immunogenicity of the YF vaccine in adults with CKD on dialysis. The vaccine was shown to be safe in this population. However, it revealed low seroconversion rates.

In healthy people, after the first YF vaccination, mild local and systemic manifestations occurred in approximately 4%. The major adverse event rates after the 17DD vaccine from Bio-Manguinhos were 0.9/100,000 doses for hypersensibility reactions, 0.08/100,000 doses for neurologic diseases, and 0.03/100,000 doses for viscerotropic diseases^6^. In our study, 5.8% of individuals experienced local manifestations and 2.7% had mild systemic manifestations. No severe VAE were observed, suggesting that the YF vaccine is safe for CKD patients on dialysis. These data align with the results of two retrospective studies, which did not identify any serious VAE in 45 patients (Facincani et al.^13^) and in 142 patients (Lara et al.^14^) with CKD undergoing dialysis. However, they found higher rates of localized VAE, at 24.4%^13^ and 12.9%^14^, respectively, which were likely associated with simultaneous vaccination. It is worth mentioning that the vaccine’s safety has also been observed in other moderately immunocompromised individuals, including HIV-positive patients, individuals with autoimmune diseases, and renal transplant recipients^15,16,17,18,19,20,21,22^.

The serological response of 38% observed in our samples contrasts with the 99% rate observed in immunocompetent individuals after 30 days^2^. Our findings also significantly differ from a seroconversion rate of 96.5% observed after a median of 13 years among 29 kidney transplant recipients vaccinated against YF before transplantation^23^. Additionally, even moderately immunocompromised individuals did not manifest seroconversion levels as low as those observed in our sample. Seroprotection rates after YF vaccine for individuals with HIV ranged from 83% to 100%^15–18^, while those with autoimmune diseases they ranged from 50% to 87%^19–21^. The 38% seroconversion rate aligns with findings from other vaccines in CKD patients on dialysis. Reported seroconversion rates in the literature range from 36% to 80% against influenza, 50% to 60% against hepatitis B, and 38% against diphtheria and tetanus^24,25^. The lower immunogenicity of the YF vaccine in patients with CKD can be attributed to the immune dysfunction resulting from CKD itself. CKD is characterized by an accelerated aging of the immune system, diminished cellular phagocytic activity, alterations in cellular recognition receptors, reduced numbers of B and T cells, and elevated levels of cytokines. Elevated cytokine levels are associated with decreased kidney clearance and increased intestinal permeability due to uremia. These alterations collectively result in diminished antibody production and more substantial declines in antibody titers compared to healthy individuals^8–10^.

It is important to emphasize that we did not encounter any studies employing PRNT with wild-type viruses. Our study conducted PRNT using both the vaccine virus and the wild strains YFV-4408 and ES-504. The ES-504/BRA/2017 strains were isolated from a howler monkey in the city of Domingos Martins, Espírito Santo, located in the southeast region of Brazil. This strain exhibited polymorphism related to viral replication, potentially accelerating the spread of an ongoing outbreak^26^. Interestingly, individuals who demonstrated adequate seroconversion with the vaccine virus exhibited similar results when tested against the wild YF strains.

Our study had several limitations. Firstly, the study population does not fully represent the entire population of individuals with CKD on dialysis in Brazil. Secondly, the retrospective study design prevented the real-time monitoring of VAE, and there may have been recall bias, which could have limited the reporting of non-serious VAE. Another limitation was that the PRNT was not carried out in the entire sample. Another intriguing analysis would be conducting PRNT over time to assess potential declines in antibody titers. This would enable us to closely monitor the duration of the immune response induced by the vaccine and evaluate the necessity for booster doses in patients with CKD on dialysis. Furthermore, it is possible that antibody detection alone may not be sufficient to fully evaluate the overall response to vaccination. Therefore, to better understand the efficacy of the YF vaccine, it would be also necessary to evaluate cellular immunity.

In conclusion, our findings support the safety of administering the YF vaccine to patients with CKD on dialysis, allowing for its use in endemic areas. However, the inconsistent ability of the vaccine to induce adequate immune responses warrants additional research into the potential efficacy of booster doses for improving serological response in this population. A comparative assessment of anti-AF neutralizing antibody titers in vaccinated individuals with and without kidney disease would offer valuable insights. Emphasizing the need for YF seroprotection before kidney transplantation in CKD patients is crucial, since the YF vaccine is contraindicated after transplantation.
