# Supplementary material for: Safety and immunogenicity of the yellow fever vaccine for patients with end-stage renal disease
Source: J Bras Nefrol. 2024 Aug 26;46(4):e20230202. doi: 10.1590/2175-8239-JBN-2023-0202en (PMC11349315; doi:10.1590/2175-8239-JBN-2023-0202en)
Supplement: Translation

# Translation: Segurança e imunogenicidade da vacina contra febre amarela para pacientes com doença renal em estágio terminal

**DOI:** 10.1590/2175-8239-JBN-2023-0202pt

**Authors:** Jesiree Iglésias Quadros Distenhreft, Dinair Couto-Lima, Cecilia Siliansky de Andreazzi, Juliana Feu Rosa Carrera Thomazini, Lauro Monteiro Vasconcellos, Aloísio Falqueto, Weverton Machado Luchi

**Affiliations:** 1 Universidade Federal do Espírito Santo, Faculdade de Medicina, Hospital Universitário Cassiano Antônio Moraes, Serviço de Nefrologia, Vitória, ES, Brasil.; 2 Instituto Oswaldo Cruz, Laboratório de Mosquitos Transmissores de Hematozoários, Rio de Janeiro, RJ, Brasil.; 3 Instituto Oswaldo Cruz, Laboratório de Biologia e Parasitología de Mamíferos Silvestres Reservatórios, Rio de Janeiro, RJ, Brasil.; 4 Universidad Complutense de Madrid, Faculdad de Ciencias Biológicas, Departamento de Biodiversidad, Ecología y Evolución, Madrid, Espanha.; 5 Clínica Nefrológica de Colatina, Colatina, ES, Brasil.; 6 Universidade Federal do Espírito Santo, Centro de Ciências da Saúde, Programa de Pós-Graduação em Doenças Infecciosas, Vitória, ES, Brasil.

**Keywords:** Insuficiência Renal Crônica, Eficácia, Anticorpos Neutralizantes, Segurança, Vacina contra Febre Amarela

## Abstract

**Introdução:**

Em dezembro de 2016, houve um surto de febre amarela (FA) silvestre em áreas não endêmicas da região sudeste do Brasil. A resposta imunológica à vacina contra FA e sua segurança em indivíduos com doença renal crônica (DRC) que vivem em regiões endêmicas de febre amarela não são totalmente compreendidas. O objetivo deste estudo foi avaliar a incidência de eventos adversos e a resposta sorológica após vacinação primária com a vacina 17DD-YF em pacientes com DRC submetidos à diálise.

**Métodos:**

Este foi um estudo de coorte retrospectivo e multicêntrico envolvendo 223 indivíduos com DRC que estavam em diálise após vacinação primária contra FA. Foram coletadas características clínicas, epidemiológicas e avaliados os eventos adversos da vacina (EAV). Cerca de 35 meses após a vacinação, a resposta sorológica foi avaliada em 71 (32%) pacientes usando testes de neutralização.

**Resultados:**

Não houve EAV grave em nenhum paciente. Reações locais foram relatadas em 13 indivíduos (5,8%), enquanto 6 (2,7%) relataram reações sistêmicas generalizadas e 205 (91,9%) não apresentaram nenhum EAV. Nenhuma característica clínica ou epidemiológica predisse a ocorrência de EAV. Uma resposta sorológica adequada foi encontrada em 38% dos participantes e nenhuma das características clínicas ou epidemiológicas foi associada à imunogenicidade.

**Conclusão:**

Os desfechos de nosso estudo sugerem que a vacina contra FA é bem tolerada em pacientes com DRC em diálise, mas não induz uma resposta imunológica adequada. Pesquisas futuras devem se concentrar na avaliação das respostas imunes tanto celulares quanto humorais após a administração de várias doses da vacina contra FA.

## Introdução

Em dezembro de 2016, teve início um surto de febre amarela (FA) silvestre em áreas não endêmicas e densamente povoadas no sudeste do Brasil, principalmente na costa. A FA é uma doença com elevada mortalidade e não há tratamento específico. A vacinação é a medida preventiva mais eficaz contra a infecção^1^. Em razão disso, foi lançada uma campanha de vacinação nas áreas afetadas.

A vacina contra a FA atualmente utilizada no Brasil é a vacina viva atenuada 17DD do Instituto de Tecnologia em Imunobiológicos da Fundação Oswaldo Cruz (Bio-Manguinhos). Cerca de 10 dias após a vacinação, pelo menos 80% das pessoas atingem títulos imunológicos adequados e, após 30 dias, mais de 99% das pessoas também alcançam esse resultado. A recomendação atual é que uma dose é suficiente para proporcionar proteção vitalícia^2^. No entanto, essa resposta pode ser reduzida em indivíduos imunocomprometidos^3^.

As reações adversas à vacina contra FA, normalmente leves, podem incluir sintomas como cefaleia, mialgia, febre baixa e desconforto no local da injeção, afetando aproximadamente 4% dos indivíduos vacinados. Embora seja geralmente segura, a vacina viva atenuada contra FA pode, em casos raros, levar a condições graves, como hipersensibilidade, doença neurológica aguda ou doença viscerotrópica associada à vacina. As contraindicações à vacina incluem crianças com menos de seis meses de idade, gestantes, indivíduos imunocomprometidos, histórico de doença do timo e indivíduos que tenham apresentado reações anafiláticas aos componentes da vacina^4,5^.

Considerando que se trata de uma vacina de vírus vivo atenuado, a vacina contra FA não é recomendada para indivíduos imunocomprometidos devido ao risco de eventos adversos maiores. Entretanto, dada a possibilidade de ocorrência urbana no Brasil, como ocorre na África, é necessário avaliar a segurança da vacina contra a FA para pessoas imunocomprometidas em áreas endêmicas. A alta densidade de infestação por *Aedes aegypti*, o aumento do número de casos de FA silvestre e a baixa cobertura vacinal no Brasil favorecem o ressurgimento da doença em ambientes urbanos^6^.

A vacina contra FA deve ser administrada a pacientes com DRC em diálise, se não houver contraindicações^7^. A imunidade na DRC é influenciada por fatores como toxinas urêmicas, desnutrição, inflamação crônica, alterações no eixo vitamina D-hormônio paratireoidiano e diálise terapêutica. A coexistência de ativação e supressão imunológica crônica na DRC pode tornar os indivíduos mais suscetíveis aos efeitos adversos da vacina contra FA e resultar em respostas vacinais comprometidas^8–10^.

Durante o surto de FA no Brasil, muitos indivíduos com DRC foram vacinados devido ao risco de FA. O objetivo deste estudo foi avaliar a ocorrência de eventos adversos e a resposta sorológica após a vacinação primária com 17DD-YF em pacientes com DRC em diálise.

## Métodos

### Desenho do Estudo

Esse estudo de coorte retrospectivo multicêntrico envolveu indivíduos com DRC que receberam a vacina contra FA enquanto estavam em terapia renal substitutiva. Entre janeiro de 2020 e agosto de 2021, selecionamos pacientes com DRC em diálise em instituições na região metropolitana de Vitória e Colatina, Espírito Santo, Brasil, que foi afetada pelo surto de FA. Todos os participantes haviam recebido anteriormente a vacinação primária com 17DD-YF (Bio-Manguinhos-FIOCRUZ). A maioria foi vacinada durante a campanha brasileira de vacinação contra a FA de 2017. Alguns indivíduos já haviam recebido a vacina antes de 2017 porque moravam ou tinham viajado para regiões endêmicas. Este estudo foi aprovado pelo Comitê de Ética em Pesquisa do Centro de Ciências da Saúde da Universidade Federal do Espírito Santo (CAAE 24851419.9.0000.5060). Todos os participantes do estudo assinaram um termo de consentimento após todas as dúvidas terem sido esclarecidas pelos pesquisadores.

A população do estudo consistiu em adultos de ambos os sexos com mais de 18 anos de idade, que foram vacinados contra a FA durante a terapia renal substitutiva (diálise peritoneal ou hemodiálise). Considerou-se somente a vacinação verificada por um cartão de vacinação.

As instituições médicas que participaram e o número de pacientes atendidos por esses serviços de terapia renal substitutiva foram: Instituto de Doenças Renais (Região Metropolitana de Vitória, 341 pacientes), Hospital Universitário Antônio Cassiano de Moraes (Vitória, 99 pacientes), Casa de Saúde de Santa Maria (Colatina, 133 pacientes) e Clínica Nefrológica de Colatina (Colatina, 250 pacientes). Os indivíduos que foram vacinados antes de se tornarem pacientes em diálise não foram incluídos neste estudo.

Todos os participantes foram entrevistados e seus cartões de vacinação e registros de exames médicos e laboratoriais do mês seguinte à vacinação foram analisados. Dessa população, amostras séricas de 71 indivíduos (32%) foram submetidas ao teste de neutralização por redução de placas de lise (PRNT, sigla em inglês).

### Avaliação de Segurança

Foram realizadas entrevistas estruturadas durante as sessões de hemodiálise ou no mesmo dia dos exames de rotina para avaliar os dados epidemiológicos e clínicos, bem como os efeitos colaterais da vacina. Além disso, os prontuários médicos dos pacientes no mês seguinte à vacinação também foram revisados.

Pacientes em terapia renal substitutiva são submetidos a uma coleta de sangue mensal, para medição de enzimas hepáticas, e anual, para sorologia de hepatite B, hepatite C e HIV. A anotação de manifestações clínicas e os resultados de exames laboratoriais estão disponíveis nos registros do serviço de diálise.

Os eventos adversos foram classificados por extensão (local versus sistêmico) e tipo (manifestações gerais, hipersensibilidade, distúrbios neurológicos e viscerotrópicos) de acordo com o Manual de Vigilância Epidemiológica de Eventos Adversos Pós-Vacinação do Ministério da Saúde do Brasil. Todos os sintomas na região onde a vacina foi aplicada foram considerados eventos adversos locais, como dor, edema, eritema, hiperemia, nódulos ou abscessos. As manifestações sistêmicas gerais incluíram febre, mialgia, cefaleia, artralgia, fraqueza, dor abdominal, náusea e tremor. Hipersensibilidade, distúrbios neurológicos e viscerotrópicos foram considerados eventos adversos maiores.

### Teste de Imunogenicidade

Em fevereiro de 2020, visando à detecção de anticorpos neutralizantes, foram coletados 5 mL de sangue periférico de 71 participantes e armazenados em tubos a vácuo sem coagulantes. O sangue foi centrifugado e o soro foi armazenado a –80 °C. As amostras foram processadas no Laboratório de Mosquitos Transmissores de Hematozoários (LATHEMA, Fundação Oswaldo Cruz – Fiocruz – RJ).

As taxas de anticorpos neutralizantes anti-FA foram avaliadas por meio do PRNT, um método de referência para avaliar a imunogenicidade pós-vacinação. Os títulos de neutralização (TN) de 1:10 e 1:20 foram utilizados como valores de corte, de acordo com estudos sobre a imunização passiva de hamsters e evidências de títulos protetores em outros arbovírus, como o vírus da encefalite japonesa^11,12^. Para este estudo, títulos acima de 1:10 foram usados para triagem e títulos acima de 1:20 foram usados como correlatos de imunidade.

Normalmente, o PRNT é realizado somente com a cepa vacinal. No entanto, criamos um modelo para comparar o vírus atenuado da vacina com o vírus selvagem. Em nosso estudo, testamos as amostras com duas cepas selvagens, além do vírus vacinal: IEC-4408 (YFV-4408) e ES-504/BRA/2017. Ambos os vírus foram isolados do soro de primatas não humanos durante epidemias no Brasil, primeiro em 2008 no Rio Grande do Sul e depois em 2017, no Espírito Santo.

Inicialmente, as 71 amostras foram submetidas à triagem utilizando a cepa vacinal 17DD. As amostras que apresentaram neutralização superior a 90% em uma diluição de ≥1:10 (indicativa de anticorpos neutralizantes detectáveis) foram submetidas a uma diluição progressiva, variando de 1:20 a igual ou superior a 1:640. Um título imunológico foi considerado adequado quando o TN foi superior ou igual a 1:20. Somente amostras com um título adequado foram posteriormente desafiadas com as cepas de tipo selvagem.

### Análise de Dados

Após a coleta de dados, foram verificadas a frequência dos eventos adversos e sua natureza e avaliadas as características que poderiam predizer a ocorrência de eventos adversos. A análise univariada foi realizada com o teste qui-quadrado para variáveis categóricas no software Past e com regressão logística simples para variáveis quantitativas no software BioEstat. Em seguida, foi realizada uma análise multivariada no software R por meio de regressões logísticas múltiplas. A análise de correspondência múltipla foi feita para exibir a associação entre fatores preditivos e a ocorrência de eventos adversos.

Para determinar se havia alguma característica preditiva para a resposta sorológica, primeiramente foi realizada uma análise de correspondência múltipla. Em seguida, as características mais significativas foram submetidas à análise multivariada no software R por meio de regressão logística múltipla. A seguir, foi realizado o teste de razão de verossimilhança.

As diversas associações foram expressas por taxas de qui-quadrado, com Z como variantes independentes e *odds ratio* determinada pelo modelo de regressão logística e respectivo intervalo de confiança de 95%. Em todos os casos, os valores de p <0,05 foram considerados estatisticamente significativos.

## Resultados

### Eventos Adversos

Um total de 223 adultos submetidos à terapia renal substitutiva participaram do estudo. A idade dos indivíduos variou de 19 a 87 anos (média, 51,13 e mediana, 52 anos); 61,9% deles eram homens. As condições subjacentes mais comuns foram hipertensão arterial (69,1%), diabetes mellitus (25,6%) e insuficiência cardíaca (5,4%). Entre os indivíduos com histórico de transplante renal, dois ainda estavam em uso de medicamentos imunossupressores (prednisona, ciclosporina e micofenolato). Além disso, quatro participantes estavam fazendo uso de medicamentos imunossupressores devido a doenças reumáticas (prednisona, ciclosporina e micofenolato). As demais características da população estudada estão descritas na Tabela 1.

**Tabela 1:** Características da população do estudo.

Características	Segurança da vacina contra FATotal (223)	Avaliação PRNTTotal (71)
Sexo masculino, n (%)	138 (61,9)	37 (52,1)
Idade, anos, média ± DP	51,1 (14,1)	51,08 (13,2)
Raça, n (%)		
Branca	89 (39,9)	23 (32,39)
Preta	67 (30,1)	20 (28,2)
Parda/multirracial	66 (29,6)	28 (39,4)
Amarela	1 (0,4)	0
Indígena	0	0
Tabagismo, n (%)	44 (19,7)	9 (12,7)
Alcoolismo, n (%)	25 (11,2)	10 (14,1)
Comorbidades, n (%)		
Hipertensão arterial	154 (69,1)	54 (76,1)
Diabetes mellitus	57 (25,6)	20 (28,2)
Câncer^a^	4 (1,8)	0 (0)
Doenças reumáticas	5 (2,2)	2 (2,8)
Insuficiência cardíaca	12 (5,4)	6 (8,4)
HIV	0	0
Hepatite C	4 (1,8)	2 (2,8)
Hepatite B	4 (1,8)	0 (2,8)
Etiologia da doença renal crônica, n (%)		
Glomerulopatias	44 (19,7)	16 (22,5)
Diabetes mellitus	47 (21,1)	18 (25,3)
Hipertensão arterial	55 (24,7)	17 (23,9)
Doença cística	8 (3,6)	5 (7,1)
Doença congênita	13 (5,8)	2 (2,8)
Outras	18 (8,1)	10 (14,1)
Desconhecida	38 (17,1)	5 (7,1)
Hemodiálise, n (%)	211 (94,6)	66 (93)
Tempo em diálise, anos, mediana (IIQ)	9,5 (6,5)	10 (4,8)
Diálise peritoneal, n (%)	12 (5,4)	5 (7,1)
Tempo em diálise, anos, mediana (IIQ)	7,2 (5)	11,9 (7)
Transplante renal anterior, n (%)	16 (7,2)	6 (8,5)
Uso de medicamentos imunossupressores^b^, n (%)	6 (2,7)	1 (1,4)

Abreviações: DP, desvio padrão; IIQ, intervalo interquartil.

Notas:

a Cânceres encontrados: pele (um), próstata (um), rim (um), reto (um).

b Prednisona, ciclosporina e micofenolato.

Entre essa população, 205 indivíduos (91,9%) não apresentaram nenhum evento adverso da vacina (EAV). Eventos adversos, classificados como manifestações locais ou sistêmicas, foram observados em 18 indivíduos (8,1%). Entretanto, nenhum indivíduo apresentou um evento adverso grave (Tabela 2).

**Tabela 2:** Eventos adversos (EA) após a vacinação contra febre amarela (FA).

EA após a vacinação	Segurança da vacina contra FA (223) n (%)
Pelo menos um EA	18 (8,1)
EA local	13 (5,8)
Dor	13 (5,8)
Edema	0
Eritema	1 (0,4)
EA sistêmico	6 (2,7)
Mialgia	2 (0,9)
Febre	3 (1,3)
Cefaleia	2 (0,9)
Mal-estar	3 (1,3)
Dor abdominal	1 (0,4)
EA grave	0
Hospitalização	1 (0,4)
Óbito	0
Níveis de ALT	218 (56,9)
0–30 U/L	4 (1,8)
31–50 U/L	1 (0,4)
51–70 U/L	

Abreviação: ALT = teste de alanina aminotransferase

Notas: Um dos 223 indivíduos apresentou EA local e sistêmico. O paciente hospitalizado apresentou febre, mal-estar, dor abdominal, mialgia e aumento dos níveis de ALT.

Um paciente sem evidência sorológica prévia de hepatite B e C apresentou manifestações clínicas de hepatite aguda, incluindo febre, mialgia e dor abdominal, juntamente com níveis elevados de ALT, duas vezes maiores que o valor de referência, no mês seguinte à vacinação. Além disso, esse indivíduo foi o único que necessitou de hospitalização, embora tenha se recuperado totalmente dos níveis elevados de transaminases em aproximadamente dois meses, sem consequências médicas duradouras.

Avaliamos as previsões para a ocorrência de eventos adversos. Na análise univariada, raça mista (χ^2^ = 16; p < 0,002) e glomerulopatia (χ^2^ = 7; p < 0,04) foram associadas a eventos adversos. Após a análise multivariada, somente a “raça mista” permaneceu como um fator de risco para a ocorrência de eventos adversos (Tabela 3).

**Tabela 3:** Fatores preditivos de eventos adversos após a vacinação em pacientes com doença renal crônica em diálise.

Características	Predição da ocorrência de fatores adversos							
	Análise Univariada				Análise Multivariada			
	N	OR	(IC95%)	p	N	OR	(IC95%)	p
Raça parda	11	4,3	1,6–11,6	0,002	223	4	1,4–10,9	0,008
Uso de medicamentos imunossupressores	2	5	0,9–27,8	0,04	223	3,4	0,5–22,7	0,21
Glomerulopatia	44	2,9	1–7,9	0,033	223	2,5	0,8–7,3	0,099

Notas: *Odds ratio* ajustado para o risco de eventos adversos pós-vacinação. A análise de regressão logística múltipla estimou que a raça mista estaria associada à ocorrência de eventos adversos após a vacinação.

A associação entre fatores preditivos e a ocorrência de EAV foi examinada por meio de análise de correspondência múltipla, conforme mostrado na Figura 1 (A e B). Foram identificadas duas dimensões que explicam coletivamente 20,5% da variação: a primeira dimensão foi responsável por 8,9% da variabilidade, enquanto a segunda explicou 11,6%. A Figura 1A mostra a correlação entre as variáveis, enquanto a Figura 1B representa cada participante individual do estudo.

**Figura 1:** Avaliação da segurança da vacina contra Febre Amarela em pacientes com Doença Renal Crônica em diálise (n = 223). **A** – Associação entre as variantes incluídas no modelo reduzido de análise de correspondência múltipla. A associação entre as características dos indivíduos é demonstrada nas duas dimensões acima. Observamos que, embora haja algum nível de agrupamento entre algumas características, nenhum grupo definido é formado em oposição. **B** – Distribuição dos indivíduos de acordo com a análise de correspondência múltipla reduzida. Os indivíduos que tiveram eventos adversos estão representados em amarelo, enquanto os indivíduos que não apresentaram eventos adversos estão em azul. Conforme demonstrado, o círculo azul está dentro do círculo amarelo, o que sugere que não existem grupos distintos.

Na Figura 1B, os indivíduos que sofreram eventos adversos são representados em amarelo, enquanto os que não sofreram são mostrados em azul. As características dos indivíduos estão correlacionadas com aquelas ilustradas na Figura 1A. Notavelmente, os indivíduos com eventos adversos estão dispersos na área do gráfico, indicando uma falta de conexão direta entre suas características e os eventos adversos. Os círculos azuis e amarelos fornecem uma aproximação da distribuição de cada grupo. É interessante notar que o círculo azul está contido dentro do círculo amarelo, o que sugere a ausência de grupos distintos.

### Imunogenicidade

O PRNT foi realizado em pacientes que se apresentaram para coleta de sangue na data proposta pelos pesquisadores, sendo 12 pacientes da região metropolitana de Vitória e 59 pacientes de Colatina. O PRNT foi realizado em 71 participantes aproximadamente 35 meses após a vacinação, com uma variação de 20 a 82 meses. Desses, 67 indivíduos (94,4%) foram vacinados em 2017, dois (2,8%) em 2018, um (1,4%) em 2013 e um (1,4%) em 2016.

Anticorpos neutralizantes detectáveis (TN ≥ 1:10) foram encontrados em 27 participantes (38%). Esses indivíduos também apresentaram títulos de neutralização adequados (TN ≥ 1:20) para todas as três cepas (vírus da vacina, Es504 e 4408). O único paciente que estava sob uso de drogas imunossupressoras não apresentou TN ≥ 1:20.

Após a análise de correspondência múltipla para identificar as categorias mais representativas (Figura 2A), realizou-se uma análise multivariada por meio de regressão logística, conforme mostrado na Tabela 4. A hipertensão arterial foi a única característica com efeito significativo na soroconversão (coeficiente = 2,1; p = 0,02), embora com baixo poder explicativo. No entanto, apesar da significância dessa variável, o modelo multivariado não forneceu muita explicação adicional, pois não diferiu significativamente do modelo nulo (teste de razão de verossimilhança; p = 0,24). Os mesmos dados são mostrados na Figura 2B, onde os indivíduos que apresentaram título de neutralização suficiente são exibidos em amarelo, enquanto os que não apresentaram estão em azul. As características desses indivíduos estão correlacionadas com as da Figura 2A. Os círculos azuis e amarelos representam uma aproximação da distribuição de cada grupo. Observamos uma sobreposição entre os dois círculos, indicando que não há uma variável estatisticamente significativa.

**Figura 2.** Avaliação da imunogenicidade da vacina contra Febre Amarela em pacientes com Doença Renal Crônica em diálise (n = 71). **A** – Associação entre as variantes incluídas no modelo reduzido de análise de correspondência múltipla. A associação entre as características dos indivíduos é evidenciada nas duas dimensões acima. Embora seja visível algum agrupamento entre algumas características, não se formam grupos definidos em oposição. **B** – Distribuição dos indivíduos de acordo com a análise de correspondência múltipla reduzida. Os indivíduos que apresentaram TN ≥1:20 estão representados em amarelo (Grupo 1), enquanto os indivíduos sem resposta sorológica (Grupo 0) são mostrados em azul. A sobreposição dos dois círculos indica a ausência de significância estatística.

**Tabela 4:** Preditores de resposta sorológica em pacientes com doença renal crônica submetidos a diálise.

Variantes	Coeficiente	Erro padrão	Taxa Z	Taxa-p
Sexo masculino	0,9	1,7	1,3	0,20
Raça preta	0,4	0,7	0,5	0,64
Raça parda ou multirracial	0,1	0,8	0,2	0,85
Transplante renal prévio	0,1	0,8	0,1	0,96
Alcoolismo	0,1	1,1	0,1	0,88
Tabagismo	0,9	1,0	1,0	0,32
HAS	2,1	0,9	2,4	0,02
DM	–0,5	0,9	–0,6	0,55
Insuficiência cardíaca	0,4	0,8	0,3	0,77
Dislipidemia	0,2	1,2	0,2	0,84
Idade	0,0	0,9	–1,3	0,18
Tempo em diálise	0,0	0,0	0,7	0,47

Abreviações: HAS: Hipertensão Arterial Sistêmica (etiologia da DRC); DM; Diabetes mellitus (etiologia da DRC).

Nota: A hipertensão arterial parece ser um fator preditivo de soroconversão (Coeficiente = 2,1; p = 0,02).

## Discussão

Neste estudo, fornecemos a primeira demonstração da segurança e imunogenicidade da vacina contra FA em adultos com DRC em diálise. A vacina mostrou-se segura nessa população. No entanto, ela revelou baixas taxas de soroconversão.

Em pessoas saudáveis, após a primeira vacinação contra a FA, ocorreram manifestações locais e sistêmicas leves em aproximadamente 4%. As taxas de eventos adversos maiores após a vacina 17DD da Bio-Manguinhos foram de 0,9/100.000 doses para reações de hipersensibilidade, 0,08/100.000 doses para doenças neurológicas e 0,03/100.000 doses para doenças viscerotrópicas^6^. Em nosso estudo, 5,8% dos indivíduos apresentaram manifestações locais e 2,7% tiveram manifestações sistêmicas leves. Não foram observados EAV graves, sugerindo que a vacina contra FA seja segura para pacientes com DRC em diálise. Esses dados estão em conformidade com os resultados de dois estudos retrospectivos, que não identificaram nenhum EAV grave em 45 pacientes (Facincani et al.^13^) e em 142 pacientes (Lara et al.^14^) com DRC submetidos à diálise. No entanto, esses estudos encontraram taxas mais elevadas de EAV locais, de 24,4%^13^ e 12,9%^14^, respectivamente, que provavelmente estiveram associadas à vacinação simultânea. Vale ressaltar que a segurança da vacina também foi observada em outros indivíduos moderadamente imunocomprometidos, incluindo pacientes HIV-positivos, indivíduos com doenças autoimunes e receptores de transplante renal^15–22^.

A resposta sorológica de 38% observada em nossas amostras contrasta com a taxa de 99% observada em indivíduos imunocompetentes após 30 dias^2^. Nossos achados também diferem significativamente de uma taxa de soroconversão de 96,5% observada após uma mediana de 13 anos entre 29 receptores de transplante renal vacinados contra a FA antes do transplante^23^. Além disso, mesmo indivíduos moderadamente imunocomprometidos não manifestaram níveis de soroconversão tão baixos quanto os observados em nossa amostra. As taxas de soroproteção após a vacina contra FA para indivíduos com HIV variaram de 83% a 100%^15–18^, enquanto para aqueles com doenças autoimunes variaram de 50% a 87%^19–21^. A taxa de soroconversão de 38% está de acordo com os achados de outras vacinas em pacientes com DRC em diálise. As taxas de soroconversão relatadas na literatura variam de 36% a 80% contra a influenza, 50% a 60% contra a hepatite B e 38% contra a difteria e o tétano^24,25^. A menor imunogenicidade da vacina contra FA em pacientes com DRC pode ser atribuída à disfunção imunológica resultante da própria DRC. A DRC é caracterizada por um envelhecimento acelerado do sistema imunológico, diminuição da atividade fagocítica celular, alterações nos receptores de reconhecimento celular, número reduzido de células B e T e níveis elevados de citocinas. Os níveis elevados de citocinas estão associados à diminuição da depuração renal e ao aumento da permeabilidade intestinal devido à uremia. Essas alterações resultam coletivamente em uma produção reduzida de anticorpos e em declínios mais substanciais nos títulos de anticorpos em comparação com indivíduos saudáveis^8–10^.

É importante enfatizar que não encontramos nenhum estudo empregando PRNT com vírus do tipo selvagem. Nosso estudo realizou o PRNT utilizando tanto o vírus da vacina quanto as cepas selvagens YFV-4408 e ES-504. As cepas ES-504/BRA/2017 foram isoladas de um macaco bugio na cidade de Domingos Martins, Espírito Santo, localizada na região sudeste do Brasil. Essa cepa exibiu um polimorfismo relacionado à replicação viral, o que pode potencialmente acelerar a disseminação de um surto em andamento^26^. Curiosamente, os indivíduos que demonstraram soroconversão adequada com o vírus da vacina apresentaram resultados semelhantes quando testados contra as cepas selvagens da FA.

Nosso estudo apresentou diversas limitações. Primeiramente, a população do estudo não representa totalmente a população integral de indivíduos com DRC em diálise no Brasil. Em segundo lugar, o desenho retrospectivo do estudo impediu o monitoramento em tempo real dos EAV, e pode ter havido um viés de memória, o que pode ter limitado o relato de EAV não graves. Outra limitação foi o fato de o PRNT não ter sido realizado em toda a amostra. Outra análise intrigante seria a realização de PRNT ao longo do tempo para avaliar potenciais declínios nos títulos de anticorpos. Isso nos permitiria monitorar detalhadamente a duração da resposta imunológica induzida pela vacina e avaliar a necessidade de doses de reforço em pacientes com DRC submetidos à diálise. Além disso, é possível que a detecção de anticorpos por si só não seja suficiente para avaliar completamente a resposta global à vacinação. Portanto, para melhor compreender a eficácia da vacina contra a FA, também seria necessário avaliar a imunidade celular.

Em conclusão, nossos achados apoiam a segurança da administração da vacina contra FA em pacientes com DRC em diálise, permitindo seu uso em áreas endêmicas. Entretanto, a capacidade inconsistente da vacina de induzir respostas imunológicas adequadas justifica pesquisas adicionais sobre a eficácia potencial de doses de reforço para melhorar a resposta sorológica nessa população. Uma avaliação comparativa dos títulos de anticorpos neutralizantes anti-FA em indivíduos vacinados com e sem doença renal ofereceria insights valiosos. É fundamental enfatizar a necessidade de soroproteção contra a FA antes do transplante renal em pacientes com DRC, uma vez que a vacina contra FA é contraindicada após o transplante.
